# CD24Fc ameliorates immune-related adverse events while preserving anti-tumor therapeutic effect

**DOI:** 10.1038/s41392-022-01030-x

**Published:** 2022-07-15

**Authors:** Mingyue Liu, Xu Wang, Xuexiang Du, Yan Zhang, Chunxia Ai, Siwen Hu-Lieskovan, Tianhong Li, Martin Devenport, Yang Liu, Pan Zheng

**Affiliations:** 1 Division of Immunotherapy, Institute of Human Virology and Department of Surgery, University of Maryland School of Medicine, Baltimore, MD 21202 USA; 2 Key Laboratory of Infection and Immunity of Shandong Province & Department of Immunology, School of Basic Medical Sciences, Shandong University, Jinan, 250012 China; 3 Shanghai Institute of Immunology, Department of Immunology and Microbiology, State Key Laboratory of Oncogenes and Related Genes, Shanghai Jiao Tong University School of Medicine, Shanghai, 200025 China; 4 Division of Oncology, Huntsman Cancer Institute, University of Utah School of Medicine, Salt Lake City, UT 84112 USA; 5 Division of Hematology/Oncology, Department of Internal Medicine, University of California Davis School of Medicine, University of California Davis Comprehensive Cancer Center, Sacramento, CA 95817 USA; 6 OncoC4, Inc, Rockville, MD 20805 USA

**Keywords:** Drug development, Immunotherapy

**Dear Editor**,

In combination, anti-CTLA-4 and anti-PD-1 mAb provide the most effective immunotherapy, although severe immune-related adverse events (irAEs) also occur at high frequency.^1^ It is urgent to develop strategies to reduce irAEs for wide-spread adoption of immune checkpoint inhibitors (ICIs).

The CD24–Siglec 10/G interaction constitutes an innate checkpoint that regulates inflammation triggered by danger-associated molecular patterns (DAMPs)^2–4^ and cancer pathogenesis.^5^ We have demonstrated that targeting this pathway can reduce inflammation in the colon,^6^ joints (US20130231464A1), central nervous system,^7^ as well as viral pneumonia.^8^ A newly completed Phase III study showed that CD24Fc significantly accelerated clinical recovery of hospitalized COVID-19 patients while reducing disease progression, including death and invasive mechanical ventilation.^9^ Correspondingly, CD24Fc attenuates COVID-19-associated systemic immunopathology.^10^ Given the potent effects of CD24Fc in regulating intractable inflammation, we evaluated its therapeutic efficacy in reducing checkpoint inhibitor-associated irAEs using human CTLA-4 knock-in (*Ctla4*^*h/h*^) and humanized NSG mouse models.

*Ctla4*^*h/h*^ mice receiving ipilimumab and anti-PD-1 Ab were treated with hIgFc control or CD24Fc on days 10, 13, 16, and 19 after birth. The body weight was monitored over time, hematologic and histopathologic alterations were measured at 6 weeks of age, as diagramed in Fig. 1a. Treatment with anti-CTLA-4 and anti-PD-1 induced substantial growth retardation, while CD24Fc treatment could significantly rescued the body weight loss (Fig. 1b). Complete blood counts (CBC) were performed to evaluate the red blood cell anemia (Fig. 1c). Combination therapy of ipilimumab and anti-PD-1 Ab resulted in significant reduction of red blood cell (RBC) and blood hematocrit (HCT), while mice received CD24Fc treatment showed normal hematopoiesis.

**Fig. 1:**
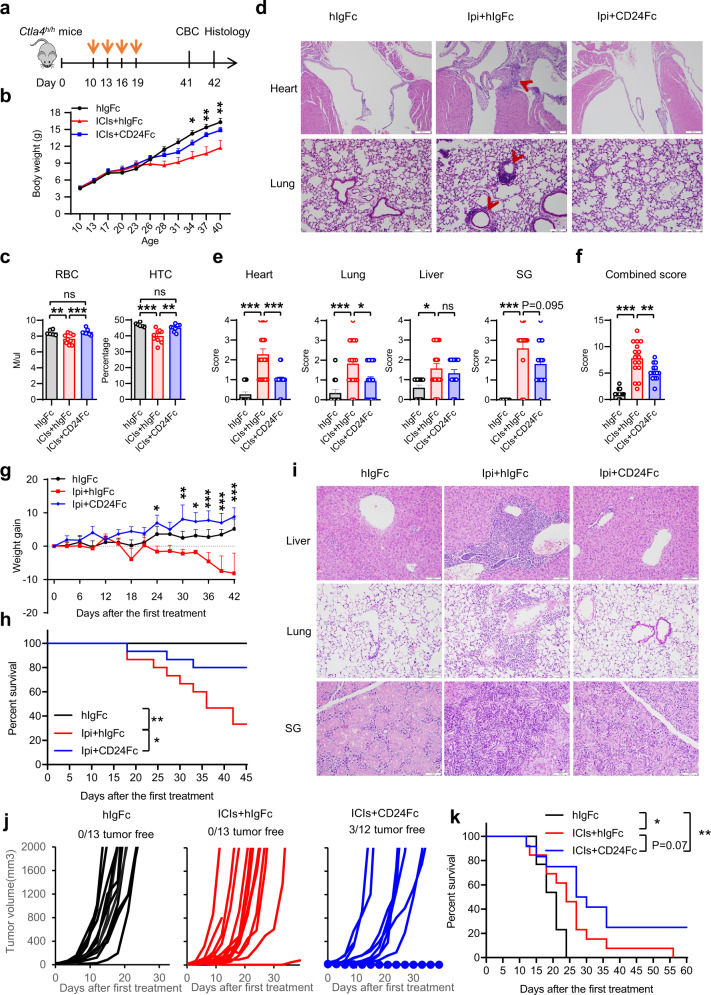
CD24Fc ameliorates irAEs while preserving anti-tumor therapeutic effect. **a**–**f** *Ctla4*^*h/h*^ KI mice were i.p. treated with 100–150 μg ipilimumab plus 100 μg anti-PD-1 Ab (RMP1-14) together with 100 μg CD24Fc or hIgFc on days 10, 13, 16, and 19. The CBC analysis was performed on day 41 after birth and necropsy was performed on day 42 after birth. **a** Timeline of drug treatment and analysis. **b** Body weight (*n* = 6–9). **c** Pure red cell aplasia was evaluated by red blood cell (RBC) and blood hematocrit (HCT). **d** Representative images of H&E-stained paraffin sections from heart and lung. Scale bar, heart 200 μm,lung 100 μm. **e** Toxicity scores of heart, lung, liver and salivary gland based on inflammation (*n* = 12–18). **f** Composite scores of all organs and glands. **g**–**i** Humanized NSG mice from the same donor of hCD34^+^ cells were treated with 100 µg ipilimumab together with 300 µg CD24Fc or hIgFc every 3 days for total four injections. **g** Body weight gain curve. One mouse from ipilimumab plus hIgFc treated group was excluded from analysis due to death on day 18. **h** Survival curve (*n* = 13–15). **i** Representative images of H&E-stained paraffin sections from liver, lung and salivary gland. Scale bar, 100 μm. **j**, **k** 1 × 10^5^ B16-F10 tumor cells were injected (s.c.) on *Ctla4*^*h/h*^ mice (*n* = 12–13) and treated (i.p.) with 200 μg Ipilimumab plus 200 μg anti-PD-1 (RMP1-14) together with 200 μg hIgFc or CD24Fc on days 8, 11, and 14. Tumor volume (**i**) and mice survival curve (**j**) is shown. Data in (**b**, **g**) were analyzed by two-way repeat measurement ANOVA with Bonferroni multiple comparison test. ICIs+hIgFc vs ICIs+ CD24Fc were compared at indicated time points. Data in (**c**), (**e**), and (**f**) were analyzed by one-way ANOVA with Bonferroni’s multiple comparisons. **h**, **k** Kaplan–Meier survival analysis. Statistical significance of the *P*-value was determined by log-rank test. Data are mean ± SEM. *p < 0.05, **p < 0.01, ***p < 0.001. Representative data of two independent experiments in (**b**, **c**, **g**) were shown, *n* = 6–9. Data in (**e**, **f**, **h**) were combined from three experiments. Those in (**j**, **k**) were combined from two experiments

To evaluate the therapeutic effect of CD24Fc on irAE, mouse organs were harvested at 1 month after the treatment and scored double-blind for inflammation. Representative tissue sections of heart and lung are shown in Fig. 1d; scores from individual mice in each group are presented in Fig. 1e and composite scores of all organs are presented in Fig. 1f. Anti-CTLA-4 and anti-PD-1 therapy-induced severe inflammation in all organs examined, while CD24Fc treatment significantly reduced the inflammatory state in heart and lung (Fig. 1d, e). A trend of decreased inflammation was also observed in liver and salivary gland after CD24Fc treatment. Liver damage was also evaluated by ALT and AST level in serum. As shown in Supplementary Fig. 1, ICIs caused high ALT level (>100) in 44% mice and high AST level (>200) in 29% mice, while the percentage is 23% and 0 in those treated with CD24Fc. When the scores from all organs were combined, the protective effect of CD24Fc against irAEs was statistically very significant (Fig. 1f).

We also evaluated the therapeutic effect of CD24Fc on irAEs in CD34^+^ humanized NSG mouse model. Successfully reconstituted humanized NSG mice were treated with 100 μg ipilimumab Ab in conjunction with hIgFc or CD24Fc every 3 days for four injections in total. Ipilimumab treatment induced significant body weight loss in the mice, which followed by progressive mortality over a month period, resulting in ~70% mortality rate. Remarkably, CD24Fc reduced the mortality to 20%, and largely prevented ipilimumab-induced weight loss (Fig. 1g, h). Histological analysis showed that CD24Fc abrogated ipilimumab-induced inflammation in multiple organs (Fig. 1i). These data demonstrated that CD24Fc conferred dramatic improvement of irAEs induced by immune checkpoint inhibitors.

A pressing issue is whether CD24Fc interferes with cancer immunotherapy. We inoculated B16-F10 tumor cells in *Ctla4*^*h/h*^ mice and treated them with ipilimumab and anti-PD-1 Ab in conjunction with hIgFc or CD24Fc. As shown in Fig. 1j, combination Ab therapy slowed tumor progressions. Notably, when combined with CD24Fc, one-quarter mice (3/12) from CD24Fc treatment group completely rejected their tumors. The survival curve supported that CD24Fc at least had no negative impact on tumor inhibition induced by checkpoint inhibitors (Fig. 1k).

To further study the role of CD24Fc, or in combination with Ipilimumab in tumor progression, we treated established B16-F10 or MC38 tumors with CD24Fc or control hIgFc. The data are presented in Supplementary Fig. 2a–c. We found that CD24Fc monotherapy moderately inhibited tumor progression (Supplementary Fig. 2a, b). Since Ipilimumab alone showed substantial tumor suppression, very little additional effect of CD24Fc was observed (Supplementary Fig. 2c). Then we performed flow cytometry to evaluate the impact of CD24Fc on tumor microenvironment (TME). Gating strategy of different FACS panel were shown in Supplementary Fig. 3a–c, CD24Fc treatment showed no effect on the amounts of tumor-infiltrating T cells or tumor-associated macrophage (TAM) (Supplementary Fig. 3d). In vitro experiment also shows that CD24Fc does not affect macrophage phagocytosis function (Supplementary Fig. 4). In the absence of ICIs, CD24Fc reduced % of Treg among CD4^+^T cells (Supplementary Fig. 3e). Ipilimumab potently reduced Treg in TME of MC38 model and masked the effect of CD24Fc (Supplementary Fig. 3e), which is consistent with the lack of additional anti-tumor effect of CD24Fc in this model (Supplementary Fig. 2c). In addition, we evaluated the T cell function by measuring the surface expression of inhibitory receptors TIM-3 and PD-1. As shown in Supplementary Fig. 3f, CD24Fc treatment decreased the expression of PD-1 and TIM-3 on CD8^+^ T cells, as well as the TIM-3 level on CD4^+^ T cells. Again, due to stronger effect of Ipilimumab in this model, no additional effect was observed when both drugs were used in combination. These data suggest CD24Fc has the potential to optimize tumor microenvironment and augment anti-tumor immunity. In transgenic mice expressing human Siglec 10 under its endogenous regulatory elements, Siglec-10 were significantly increased in TME (Supplementary Fig. 5). Interestingly, Tregs express higher level of Siglec-10 than conventional CD4^+^ T cells in both spleen and TME.

As the first step to understand how CD24Fc reduces Treg in tumor microenvironment, we tested if CD24Fc affect Treg differentiation in the presence of TGFβ and other inflammatory cytokines. As shown in Supplementary Fig. 6, CD24Fc did not affect Tregs differentiation in vitro in the presence of added cytokines, which suggests that CD24Fc may not directly affect Tregs differentiation from naïve T cells if exogenous cytokines are provided. It is possible that the effect of CD24Fc on Treg frequency in TME relates to its repression of inflammatory cytokines.^10^

Taken together, data presented herein revealed therapeutic effect of CD24Fc on irAEs, induced by Ipilimumab monotherapy or combined with anti-PD-1 Ab, without adversely affecting the cancer immunotherapeutic effects of the ICIs. Further studies are needed to fully elucidate the mechanism of action by CD24Fc. Our results provide a potential therapeutic strategy for irAEs by targeting the CD24–Siglec innate immune checkpoint.

## Supplementary information

Supplementary Materials

## Data Availability

All materials are available in the main text or supplementary materials. Further information and requests for resources and reagents should be directed to and will be fulfilled by the corresponding author.
