# Supplementary material for: CD24Fc ameliorates immune-related adverse events while preserving anti-tumor therapeutic effect
Source: Signal Transduct Target Ther. 2022 Jul 15;7:224. doi: 10.1038/s41392-022-01030-x (PMC9283527; doi:10.1038/s41392-022-01030-x)
Supplement: Supplementary file 1 — Supplementary Materials [file 41392_2022_1030_MOESM1_ESM.docx]

Supplementary Materials for

CD24Fc ameliorates immune-related adverse events while preserving anti-tumor therapeutic effect

Mingyue Liu, Xu Wang, Xuexiang Du, Yan Zhang, Chunxia Ai, Siwen Hu-Lieskovan, Tianhong Li, Martin Devenport, Yang Liu & Pan Zheng

Correspondence to: [yangl@oncoc4.com](mailto:yangl@oncoc4.com) and pzheng@oncoc4.com

**This PDF file includes:**

Materials and Methods

Figures. S1 to S6

Materials and Methods

**Animals**

C57BL/6 mice that express the CTLA-4 protein with 100% identity to human CTLA-4 protein under the control of endogenous mouse *Ctla4* locus have been previously described^1^. NSG mice were purchased from Jackson Laboratories. Transgenic mice carrying the human transgenes for Sigelc-10 were generated by injecting BAC containing the upstream and downstream regulatory sequence of human Siglec-10 gene into single cell stage fertilized eggs from C57BL/6 mice (Cyagen Biosciences Inc.). Siglec-10 Tg mice were then mated with Siglec-G KO mice to obtain Siglec-10/Siglec-G KO mice. All mice were maintained at the Research Animal Facility of Children’s Research Institute at the Children’s National Medical Center or the Institute of Human Virology at the University of Maryland Baltimore School of Medicine. All studies involving mice have been approved by the Institutional Animal Care and Use Committee.

**Cell culture and treatment**

The murine colon tumor cell line MC38, melanoma cell line B16-F10 and human breast cancer cells MCF-7 were purchased from ATCC. The cells were grown in DMEM (Dulbecco’s Modified Eagle Medium, Gibco) supplemented with 10% FBS (Hyclone), 100 units/mL of penicillin and 100 μg/mL of streptomycin (Gibco). All cell lines were incubated at 37 °C and were maintained in an atmosphere containing 5% CO2.

**Fusion protein and Antibodies**

CD24Fc is a recombinant fusion protein consisting of the extracellular domain of mature human CD24 linked to the human immunoglobulin G1 (IgG1) Fc domain and provided by OncoImmune, Inc. Recombinant Ipilimumab with amino acid sequence disclosed in WC500109302 and http://www.drugbank.ca/drugs/DB06186 was provided by Lakepharma Inc. (San Francisco, CA, USA) and Alphamab Inc. (Suzhou, Jiangsu, China). Clinical Ipilimumab was also used for some of the studies. Mouse anti-PD-1 antibody (RMP1-14) was purchased from Bioxcell Inc. (West Lebanon, NH, USA). Azide-free human IgG-Fc was purchased from Athens Research and Technology (Athens, GA, USA).

***Ctla4^h/h^* irAE model and histopathology analysis**

Young *Ctla4^h/h^* mice were treated (i.p.) with 100-150 μg Ipilimumab plus 100 μg anti-PD-1 Ab (RMP1-14) together with 100 μg CD24Fc or hIgFc on day 10, 13, 16 and 19 after birth. To avoid cage variation, mice in the same cages were individually tagged and separated to different groups based on body weight. Organs were harvested on day 42 and fixed in 10% formalin. Hematoxylin and eosin (H&E) staining was performed by Histoserv, Inc (Germantown, MD, USA). Inflammation status of those organs were scored double blind. Score criteria has been described previously^2^. Briefly, score criteria: heart, infiltration in pericardium, right or left atrium, base of aorta, and left or right ventricle each count as 1 point; lung scoring is based on lymphocyte aggregates surrounding bronchiole, 1 stands for 1–3 small foci of lymphocyte aggregates per section, 2 stands for 4–10 small foci or 1–3 intermediate foci, 3 stands for more than 4 intermediate or presence of large foci, 4 stands for marked interstitial fibrosis in parenchyma and large foci of lymphocyte aggregates; liver scoring is based on lymphocyte infiltrate aggregates surrounding portal triad, 1 stands for 1–3 small foci of lymphocyte aggregates per section, 2 stands for 4–10 small foci or 1–3 intermediate foci, 3 stands for 4 or more intermediate or the presence of large foci, 4 stands for marked interstitial fibrosis in parenchyma and large foci of lymphocyte aggregates; Salivary gland scoring is based on lymphocyte infiltration in submandibular gland: 1 stands for 1–3 small foci of lymphocyte aggregates per section, 2 stands for 4–10 small foci or 1–3 intermediate foci, 3 stands for 4 or more intermediate or presence of large foci, 4 stands for marked interstitial fibrosis and tissue destruction in parenchyma and large foci of lymphocyte aggregates.

**Complete blood counts**

Blood samples (50 μL) were collected when mice were 41-44 days old by using tubes with K2EDTA (BD) and analyzed by HEMAVET HV950 (Drew Scientific Group, Miami Lakes, FL, USA) following the manufacture’s manual.

**Liver function assay**

Liver functions were evaluated in mice by determining the serum ALT and AST activity using ALT activity assay kit (RANDOX, Cat. AL1205) and AST activity assay kit (RANDOX, Cat. AS1204), according to the manufacturer’s instruction.

**Human CD34^+^ stem cell reconstituted NSG mice irAE model**

0.5-1×10^5^ human CD34^+^cord blood stem cells were injected intrahepatically into 0 to 2 days old NSG pups or were injected intravenously into 4-8 weeks old age adult NSG mice that had been sub-lethally irradiated (1 Gy). The reconstitution of these mice with human CD45^+^ (hCD45^+^) cells was determined in peripheral blood at 12-20 weeks by flow cytometry. Humanized NSG mice were treated, respectively, with indicated antibodies every 3 days for total four injections. To avoid donors' variation, the humanized NSG mice derived from the same donor were used in each independent experiment. For survival analyses, mice are considered to have reached endpoint if they become moribund or died.

**Tumor model and treatment**

MC38 (5-8×10^5^) or B16-F10 (1-2×10^5^) were injected subcutaneously (s.c.) into the flank of mice. For CD24Fc monotherapy, B16 tumor-bearing mice were treated (i.p.) with 100 μg control hIgGFc or CD24Fc on days 3, 6, and 9; MC38 tumor-bearing mice were treated with 100 μg hIgFc or CD24Fc on day 10, 13 and 16. For combination therapy, immunotherapies were initiated on day 8 after tumor inoculation with indicated doses. Tumor volumes were measured by length (a) and width (b) and calculated as tumor volume = ab^2^/2. Mice with tumor volumes less than 2000 mm^3^ were considered to be surviving.

**Flow cytometry**

MC38-bearing mice were i.p. treated with 100 μg hIgFc or CD24Fc on day 8, 11 and 14. Tumor masses were collected on day 16, minced into small pieces and digested with 1 mg/mL collagenase IV (Sigma-Aldrich, cat# C5138), 0.1 mg/mL Hyaluronidase (Sigma-Aldrich, cat# H6254) and 0.01 mg/mL deoxyribonuclease I (Sigma-Aldrich, cat# D5025), washed and filtered through 100 μm cell strainer. Single-cell suspensions were blocked with anti-FcR (clone 2.4G2, BioXcell) and then stained with antibodies for flow cytometry. Intracellular staining was performed with Intracellular Fixation and Permeabilization kit (Cat# 88-8824, eBioscience) according to the manufacturer’s instructions. The samples were analyzed by the BD Canton II Flow cytometer and data were analyzed by Flowjo software. All CD45^+^ cell populations were gated for single cells in forward scatter area (FSC-A) versus height (FSC-H) and side scatter area (SSC-A) versus height (SSC-H) plots.

**Phagocytosis assay**

Human monocytes were isolated from peripheral blood by adherence and cultured in complete RPMI-1640 medium supplemented with 50 ng/ml M-CSF for 4-5 days. Then M2 macrophage were induced by 50 ng/ml TGFβ1 and IL10 for 3 days. MCF-7 cells were labeled with 2 μM CellTracker™ Violet Dye (Invitrogen, cat# C10094) for ten minutes. Macrophages were digested by Accutase Cell Detachment Solution (Biolegend, cat# 423201). 5×10^4^ macrophages and 1×10^5^ labeled MCF-7 cells were cocultured in triplets in ultra-low attachment U-bottom 96-well plate in the presence of hIgFc or CD24Fc of indicated concentration for 2 hours. Cells were collected and stained with CD11b-APC-Cy7 and fixed with 2% paraformaldehyde for further flow cytometry analysis.

**Treg differentiation**

Human naïve CD4^+^ T cells were isolated from human PBMCs using the MojoSort™ Human CD4 Naïve T Cell Isolation Kit (Biolegend, Cat# 480041). Seed 5×10^4^ cells in triplets to a human CD3 antibody-coated plate with human treg differentiation media (R&D, Cat#CDK006) in the presence of indicated concentration of hIgFc or CD24Fc. Incubate the cells in a 37 °C, 5% CO2 humidified incubator for 5 days.

**Statistical analysis**

The specific tests used to analyze each set of experiments are indicated in the figure legends. For each statistical analysis, appropriate tests were selected on the basis of whether the data were normally distributed by using the Shapiro–Wilk test. Data were analyzed using an unpaired two-tailed Student’s *t* test or Mann–Whitney test to compare between two groups, either one-way or two-way ANOVA (analysis of variance) with Sidak’s correction for multiple comparisons, two-way repeated measures ANOVA for behavioral tests. In the graphs, y-axis error bars represent S.E.M. Statistical calculations were performed using GraphPad Prism software (GraphPad Software, San Diego, California).

**References:**

1. Lute, K.D.*, et al.* Human CTLA4 knock-in mice unravel the quantitative link between tumor immunity and autoimmunity induced by anti-CTLA-4 antibodies. *Blood* **106**, 3127-3133 (2005).

2. Du, X.*, et al.* Uncoupling therapeutic from immunotherapy-related adverse effects for safer and effective anti-CTLA-4 antibodies in CTLA4 humanized mice. *Cell Res* **28**, 433-447 (2018).

Figure. S1.

*****

P=0.08

Fig S1. CD24Fc decreased liver damage induced by ICIs treatment. *Ctla4^h/h^* KI mice were i.p. treated with 100-150 μg ipilimumab plus 100 μg anti-PD-1 Ab (RMP1-14) together with 100 μg CD24Fc or hIgFc on day 10, 13, 16 and 19. Sera were collected on day 42. ALT and AST levels were shown. Data are mean ± SEM and analyzed by one-way ANOVA with Bonferroni’s multiple comparisons. *p < 0.05, **p < 0.01, ***p < 0.001. Data in were combined from two experiments (n=8-18).

Figure. S2.

a b

***

B16

MC38

***

**c**

Fig S2. CD24Fc inhibited tumor progression. 1x10^5^ B16 tumor cells were injected (s.c.) into *Ctla4^h/h^* mice (n=6) and treated (i.p.) with 100 μg control hIgFc or CD24Fc on days 3, 6, and 9. (b) MC38-bearing mice were i.p. treated with 100 μg hIgFc or CD24Fc on day 10, 13 and 16. (c) MC38 tumor-bearing *Ctla4^h/h^* mice were treated (i.p.) with 100 μg control hIgGFc or CD24Fc, 10 μg Ipilimumab plus 100 μg hIgFc or CD24Fc, on days 10, then treated with 100 μg control hIgFc or CD24Fc on day13 and 16. Data were analyzed by two-way repeat measurement ANOVA with Bonferroni multiple comparison test. hIgFc vs CD24Fc were compared at indicated time points. *p < 0.05, **p < 0.01, ***p < 0.001. Data are mean ± SEM. Representative data of two independent experiments were shown.

Figure. S3.

FSC-A

SSC-A

CD45

SSC-A

FSC-A

FSC-H

SSC-A

SSC-H

**a**


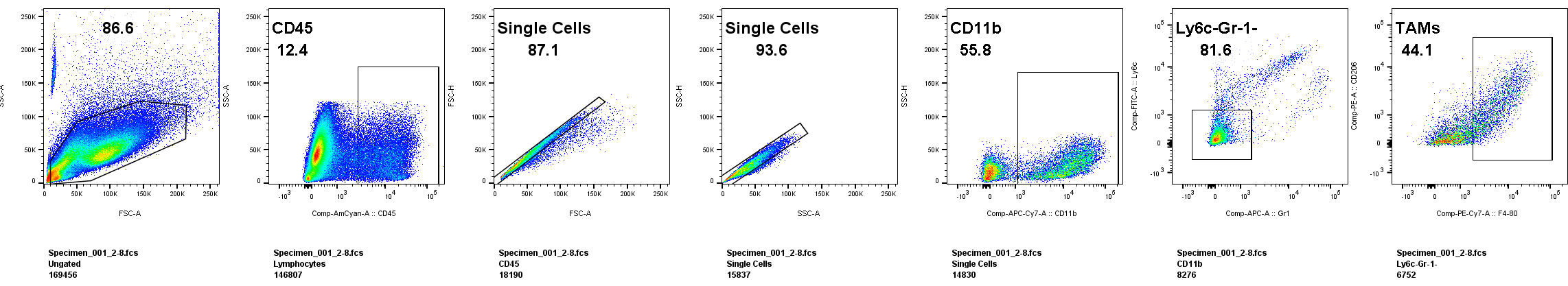

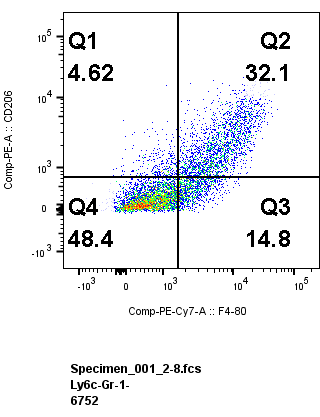

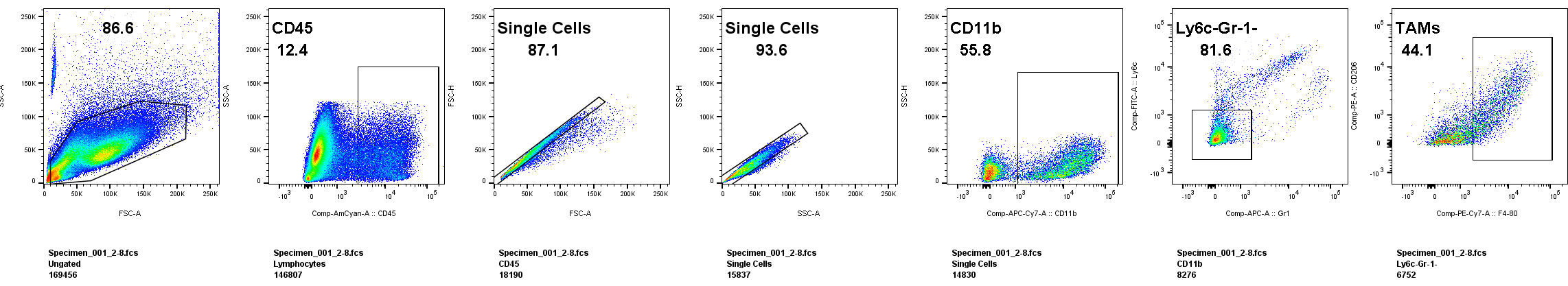

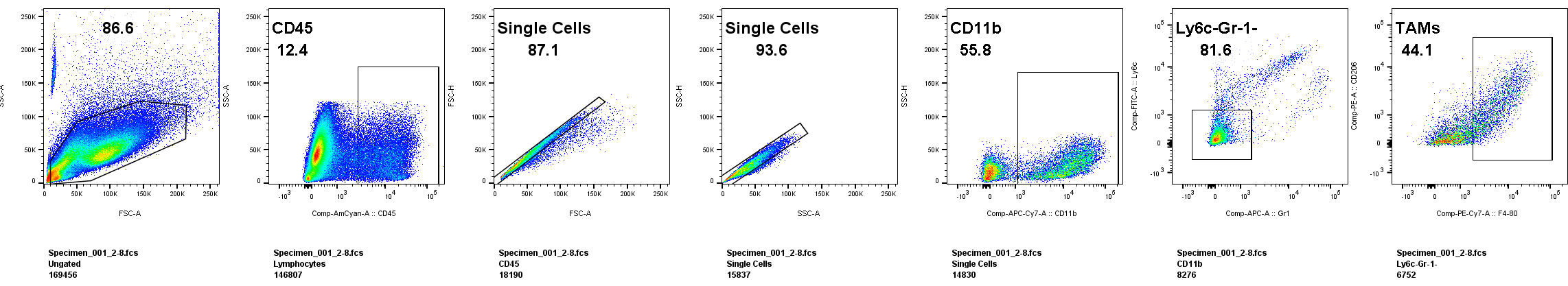


CD11b

SSC-H

Gr-1

Ly6c

F4/80

CD206

F4/80

CD206

**b**

**c**


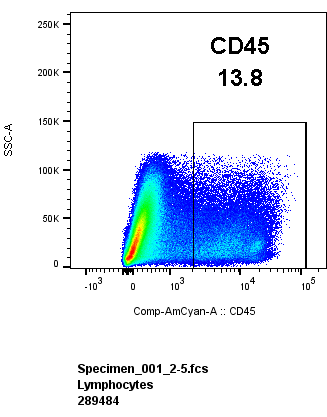

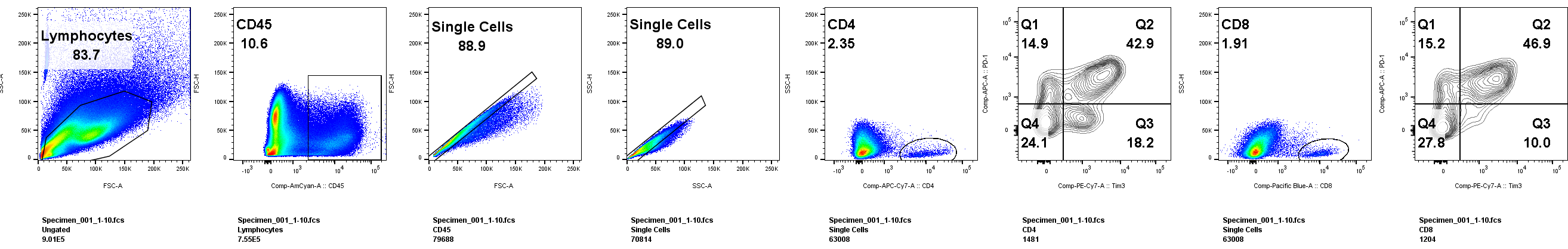

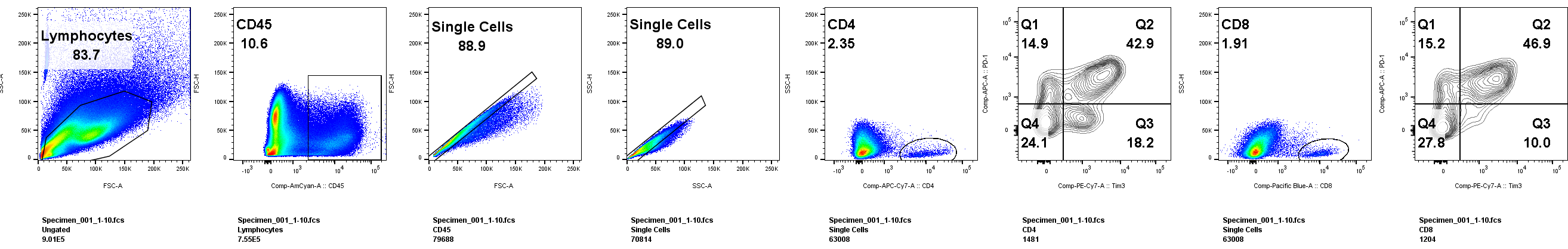

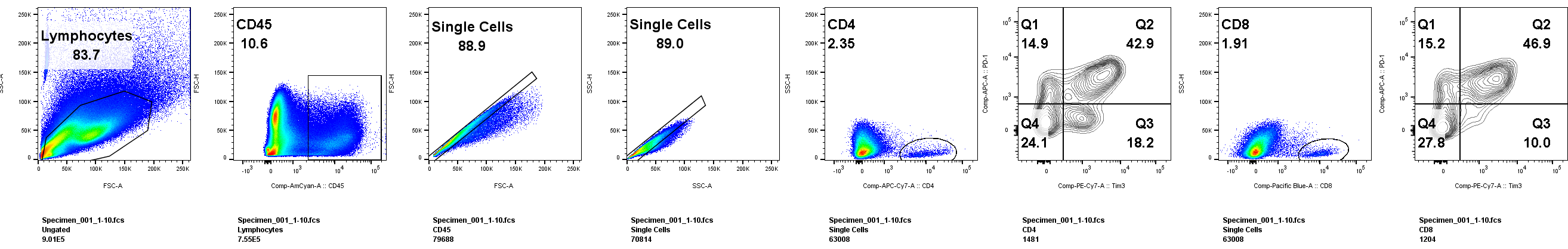

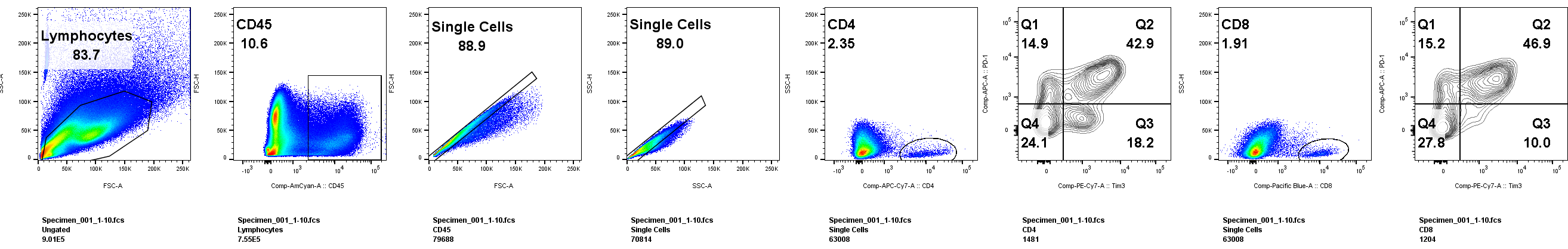

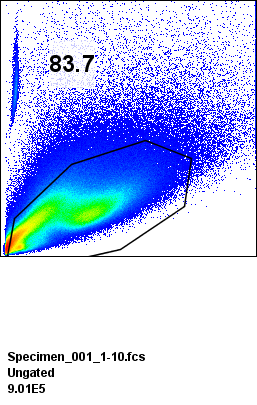

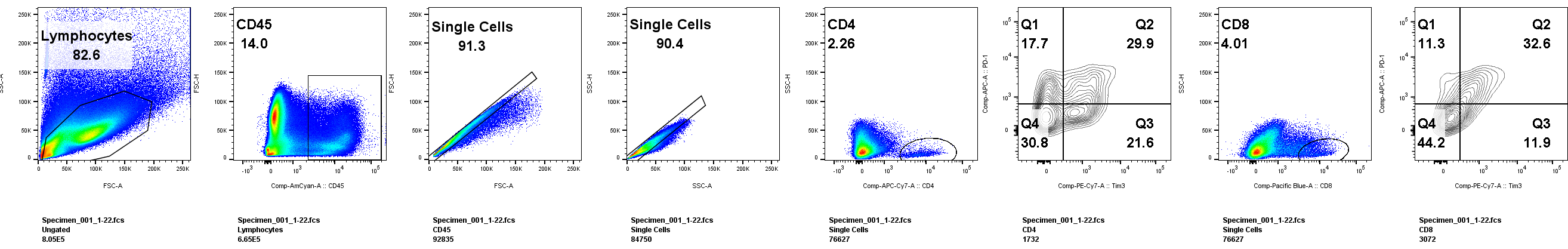


CD4

SSC-H

Tim3

PD-1

CD8

SSC-H

Tim3

PD-1


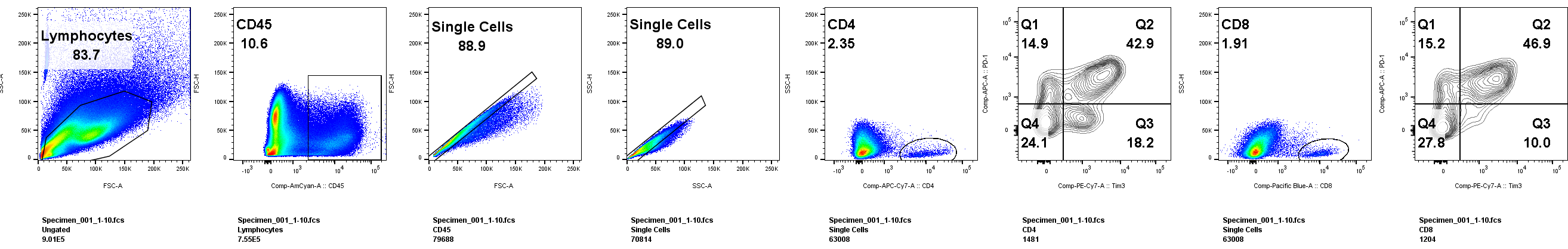

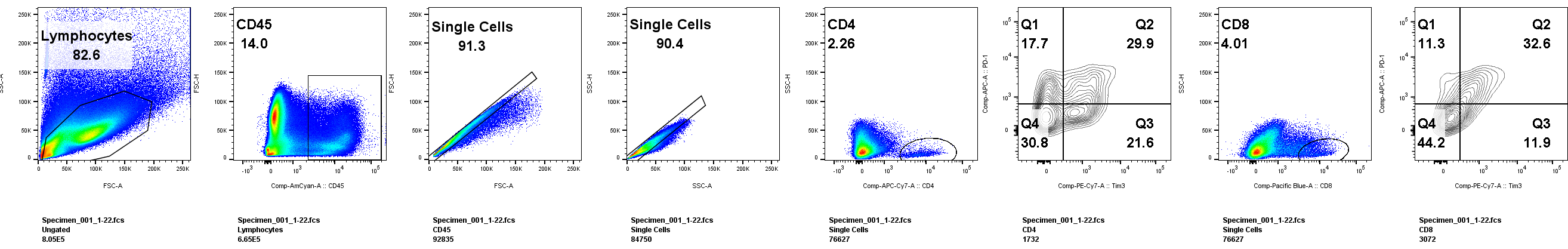

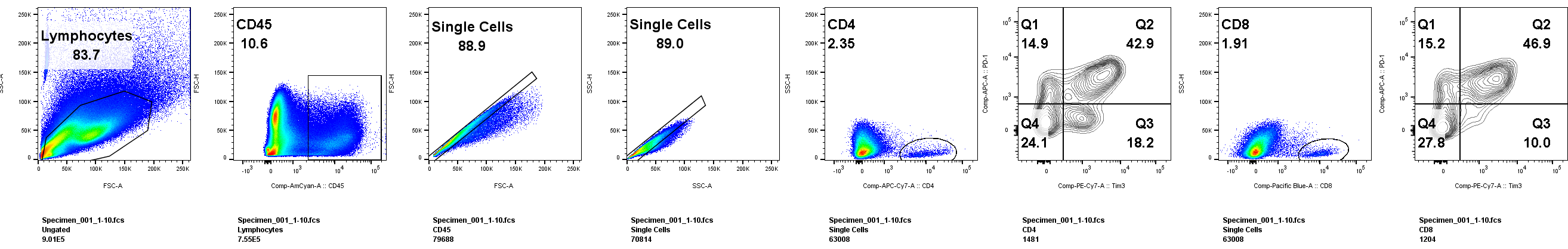


SSC-A

FSC-A

CD45

FSC-H

SSC-A

SSC-H

CD25

SSC-A

CD4

Foxp3


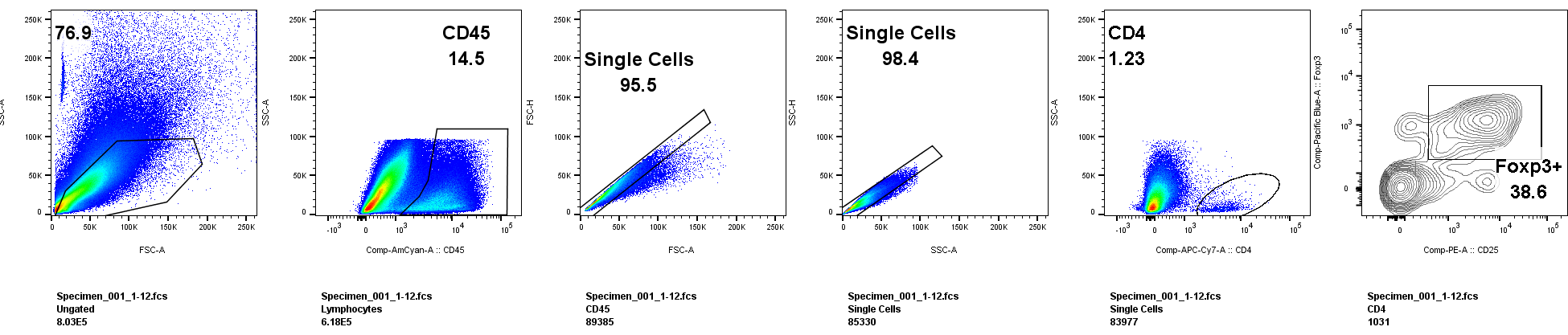

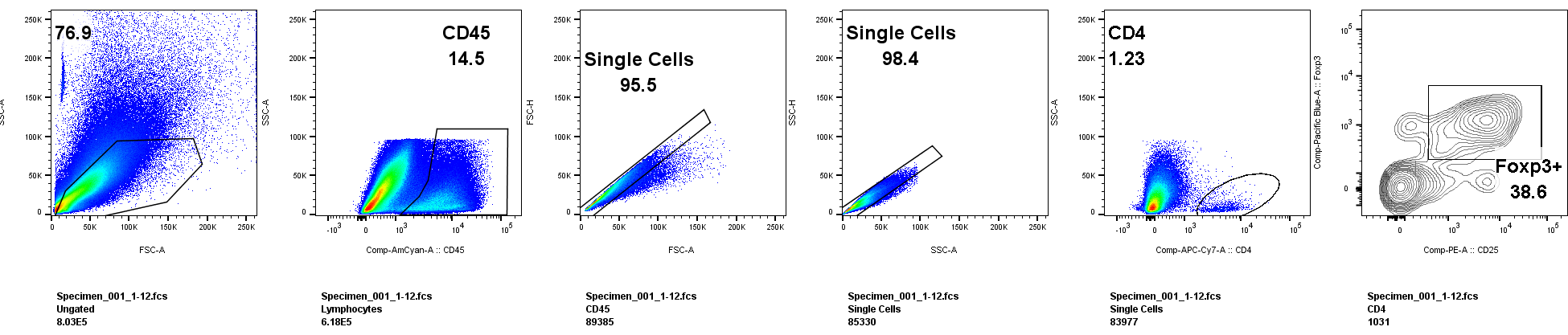

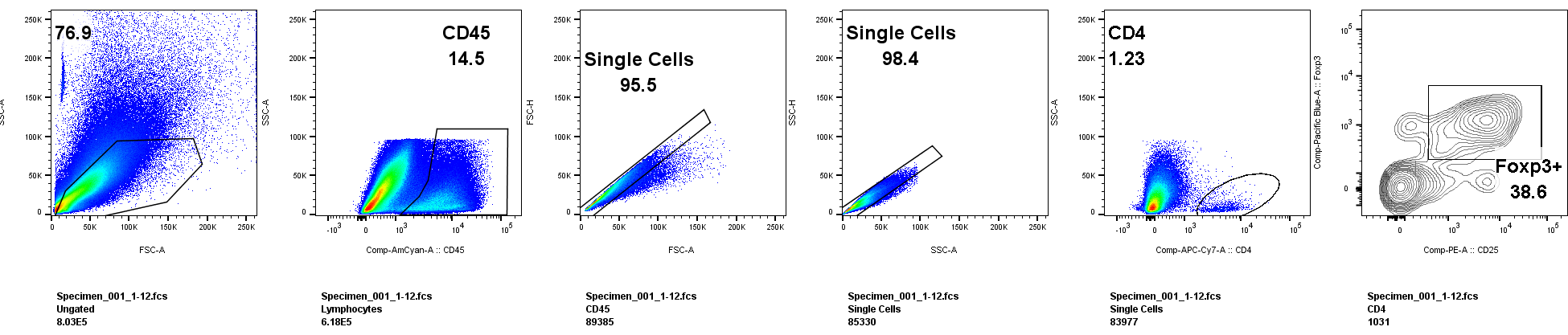

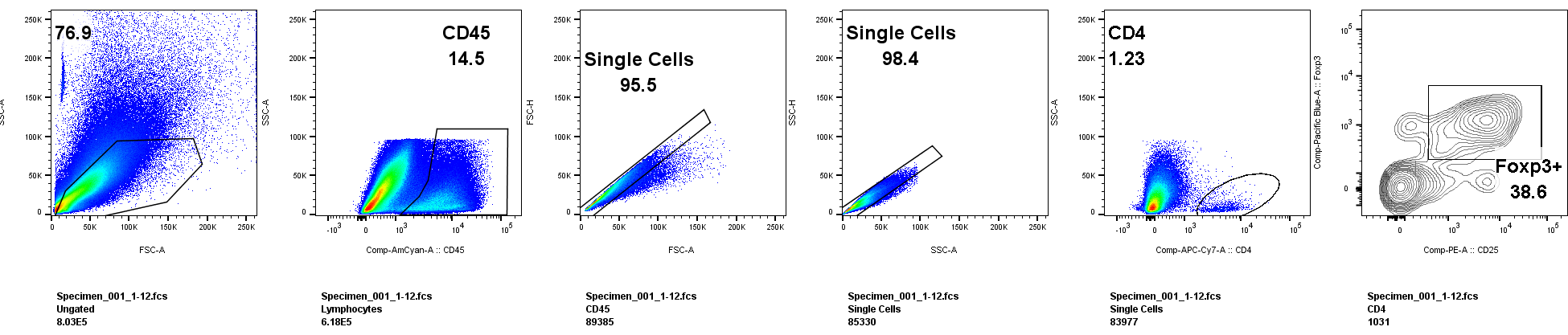

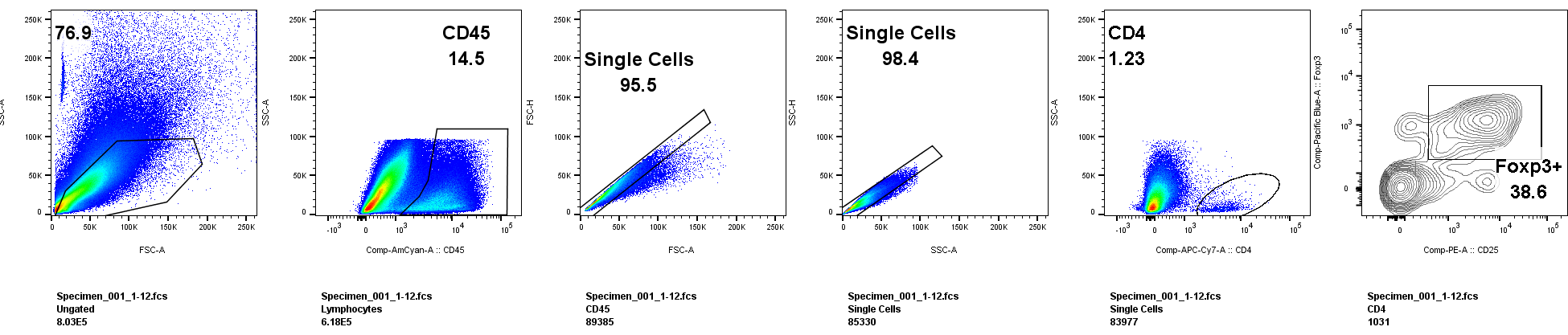


CD8

TAM

CD4

M2

**d**

**f**

**e**

Treg

CD8

CD4

P=0.08

*

***

***

*

*

*

*

*

*

**

**

Fig S3. CD24Fc decreased Treg percentage and PD-1 and TIM-3 expression on T cells in tumor microenvironment. MC38-bearing mice were i.p. treated with 100 μg hIgFc or CD24Fc on day 8, 11 and 14. Tumor masses were collected on day 16 for flow cytometry analysis. (a-c) Gating strategy for CD4^+^, CD8^+^ T cells (a), TAMs (b) and Tregs (c). (d) CD8^+^, CD4^+^ and TAM density. TAM were gated on CD45^+^CD11b^+^Gr-1^-^Ly6c^-^F4/80^+^ cells. M2-macrophage were gated on CD206^+^ cells of TAM. (e) Percentage of Tregs in CD4^+^ T cells. (f) Mean fluorescence intensity (MFI) of PD-1 and TIM-3 expression on CD8^+^ and CD4^+^ T cells. Data were analyzed by one-way ANOVA with Bonferroni’s multiple comparisons. Data are mean ± SEM. *p < 0.05, **p < 0.01, ***p < 0.001. Data were combined from two experiments, n=11.

Figure. S4.

**a**


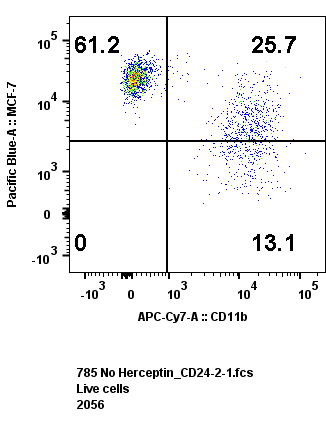

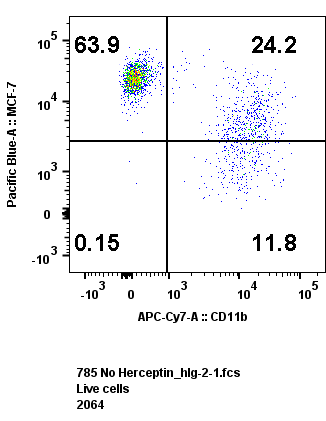

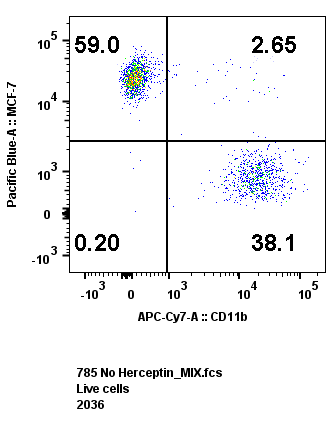


Staining control

CD24Fc

hIgFc

CD11b

Cell Trace Violet

**b**

Fig S4. CD24Fc doesn’t affect macrophage phagocytosis. Human monocytes isolated from peripheral blood were cultured with RPMI-1640 medium supplemented with 50ng/ml M-CSF for 4 days. Then M2 macrophage were induced by 50ng/ml TGFβ1 and IL10 for 3 days. MCF-7 cells were labeled with CellTracker™ Violet Dye (CTV) and cocultured with donor-derived macrophages in the presence of hIgFc or CD24Fc of indicated concentration for 2 hours. Cells were stained with CD11b-APC-cy7 and detected by flow cytometry. (a) Representative FACS profiles depicting MCF-7 phagocyted by macrophages. Single macrophage or MCF-7 were mixed after fixation and used as gating control (left panel). (b) Percentage of phagocytosis were calculated by CD11b^+^CTV^+^/CD11b^+^ × 100%. Data are mean ± SEM. Data are representative of three donors.

Figure. S5.


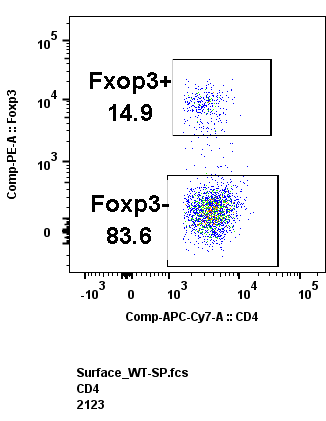

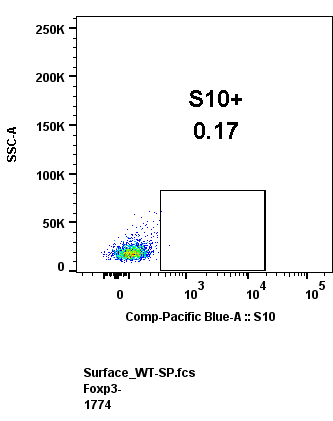

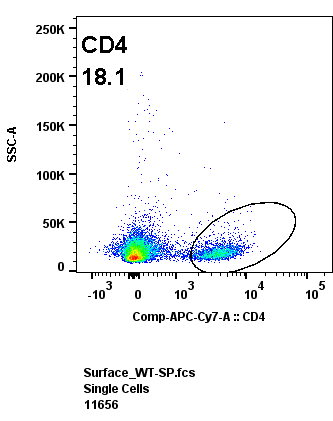

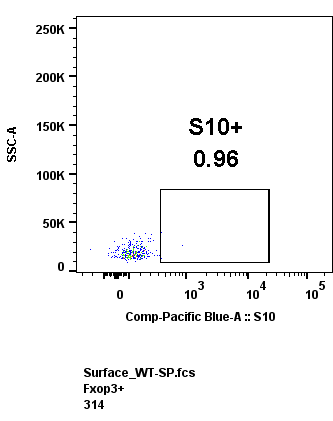

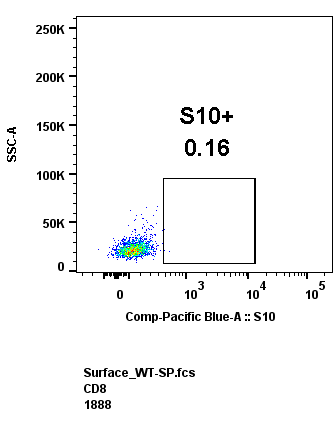

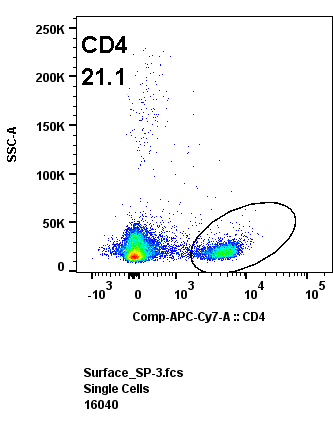

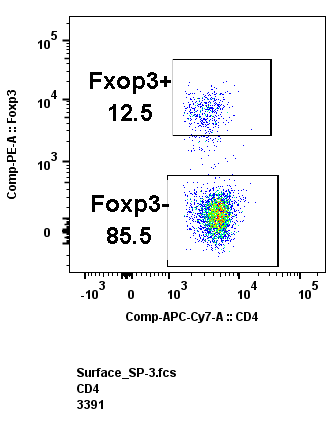

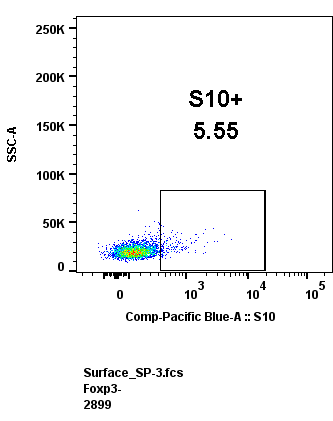

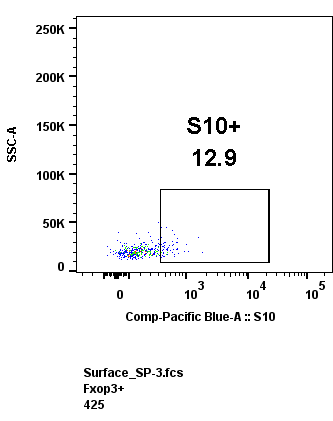

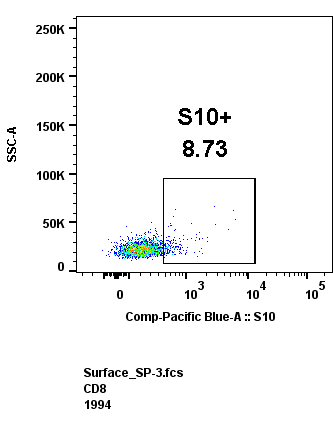

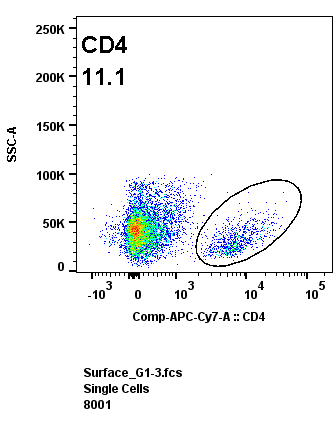

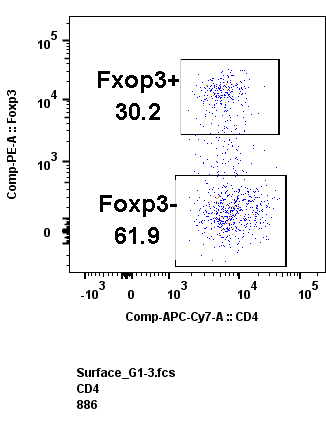

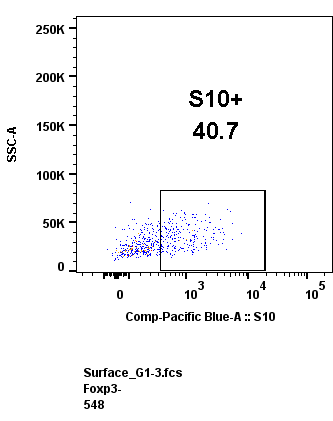

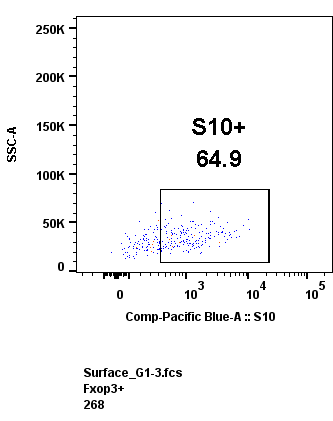

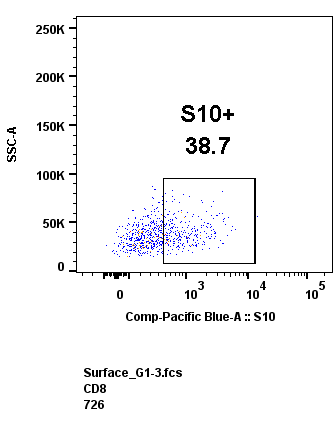

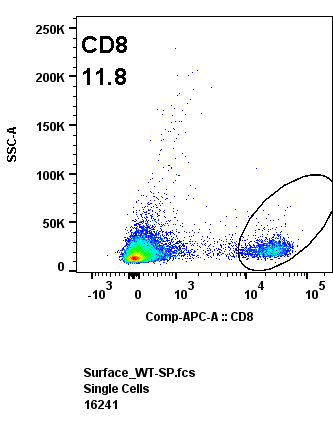

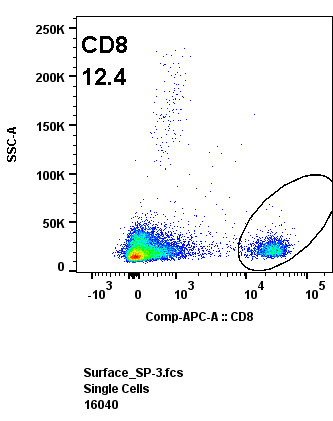

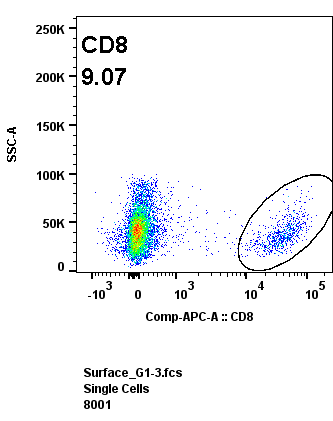


CD4

SSC-A

CD4

Fxop3

Siglec-10

SSC-A

Siglec-10

SSC-A

CD8

SSC-A

Siglec-10

SSC-A

Fxop3^-^ CD4

Fxop3^+^ CD4

CD8

**a**

WT SP

S10Tg SP

S10Tg Tumor

**b c**

***

***

***

***

*

*

Fig S5. Siglec-10 expression on T cells in splenocytes and TILs from tumor-bearing mice (n=5-6). 8x10^5^ MC38 tumor cells were injected (s.c.) into Siglec-10 transgenic/Siglec G KO mice. Tumors and spleens were collected for FACS staining on Day 17. Splenocytes form WT mice were used as siglec-10 staining negative control. (a) Representative FACS profiles depicting siglec-10 expression on Foxp3^-^, Foxp3^+^ CD4 T cells and CD8 T cells. CD4 and CD8 T cells are gated on CD45^+^ singlets. (b-c) Summary data on percentage of siglec-10 positive cells (b) and mean fluorescence intensity (MFI) of siglec-10 expression (c) among Foxp3^-^, Foxp3^+^ CD4 T cells and CD8 T cells. Data were analyzed by one-way ANOVA with Bonferroni’s multiple comparisons. Data are mean ± SEM. *p < 0.05, **p < 0.01, ***p < 0.001.

Figure. S6.

CD25

Foxp3

CTLA-4

CD127

CD25

FMO


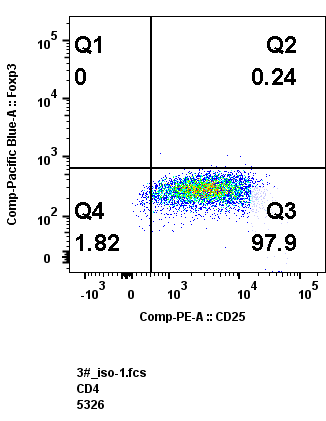

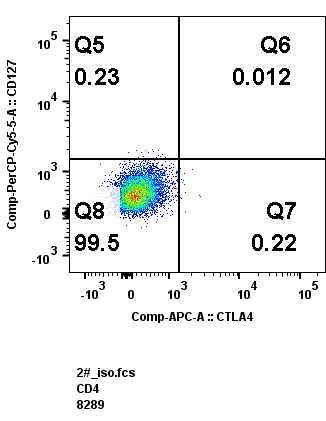

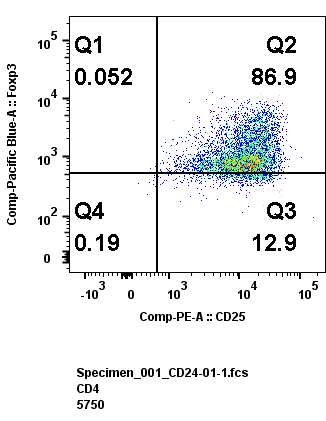

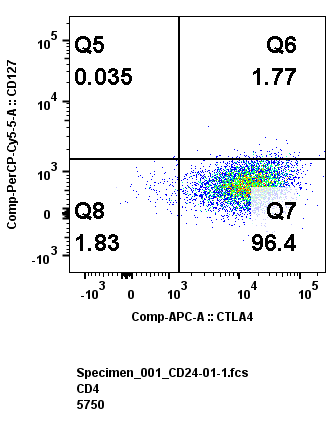


FMO

CD127

FMO controls

Treg staining

**a**

**b**

**c**

**d**

**e**

Fig S6. CD24Fc doesn’t affect Treg differentiation *in vitro*. Human peripheral naïve CD4^+^ T cells were incubated with reagents included in the Human Treg Cell Differentiation Kit in the presence of indicated concentration of hIgFc or CD24Fc for 5 days. Treg markers were stained and detected by flow cytometry. (a) Representative FACS profiles depicting Treg markers. (b) Treg number. (c) Treg percentage. (d, e) Mean fluorescence intensity (MFI) of Foxp3 (d) and CTLA-4 (e). Data are representative of three donors.
